# Supplementary material for: Direct observation of twisted stacking domains in the van der Waals magnet CrI3
Source: Nat Commun. 2024 Jul 15;15:5925. doi: 10.1038/s41467-024-50314-z (PMC11251270; doi:10.1038/s41467-024-50314-z)
Supplement: Supplementary file 1 — Supplementary Information [file 41467_2024_50314_MOESM1_ESM.pdf]

**Supplementary Information for**

# Direct observation of twisted stacking domains in the van der Waals magnet $CrI_3$

Myeongjin Jang[1,2,†], Sol Lee[1,2,†], Fernando Cantos-Prieto[3], Ivona Košić[3], Yue Li[4], Arthur R. C. McCray[4,5], Min-Hyoung Jung[6], Jun-Yeong Yoon[1,2], Loukya Boddapati[7], Francis Leonard Deepak[7], Hu Young Jeong[8,9], Charudatta M. Phatak[4,10], Elton J. G. Santos[11,12,13,*], Efrén Navarro-Moratalla[3,*] and Kwanpyo Kim[1,2,*]

[1]Department of Physics, Yonsei University, Seoul 03722, Republic of Korea.

[2]Center for Nanomedicine, Institute for Basic Science (IBS), Seoul 03722, Republic of Korea.

[3]Instituto de Ciencia Molecular, Universitat de València, Calle Catedrático José Beltrán Martínez 2, 46980, Paterna, Spain.

[4]Materials Science Division, Argonne National Laboratory, Lemont, Illinois 60439, USA.

[5]Applied Physics Program, Northwestern University, Evanston, Illinois 60208, USA.

[6]Department of Energy Science, Sungkyunkwan University (SKKU), Suwon 16419, Republic of Korea.

[7]Nanostructured Materials Group, International Iberian Nanotechnology Laboratory, Avenida Mestre Jose Veiga s/n, Braga 4715-330, Portugal.

[8]Graduate School of Semiconductor Materials and Devices Engineering, Ulsan National Institute of Science and Technology, Ulsan 44919, Republic of Korea.

[9]UNIST Central Research Facilities, Ulsan National Institute of Science and Technology, Ulsan 44919, Republic of Korea.

[10]Department of Materials Science and Engineering, Northwestern University, Evanston, Illinois 60208, USA.

[11]Institute for Condensed Matter Physics and Complex Systems, School of Physics and Astronomy, The University of Edinburgh, Edinburgh EH9 3FD, United Kingdom.

[12]Higgs Centre for Theoretical Physics, The University of Edinburgh, Edinburgh EH9 3FD, United Kingdom.

[13]Donostia International Physics Center (DIPC), 20018, Donostia-San Sebastián, Basque Country, Spain.

*esantos@ed.ac.uk, efren.navarro@uv.es, kpkim@yonsei.ac.kr

†These authors contributed equally to this work.

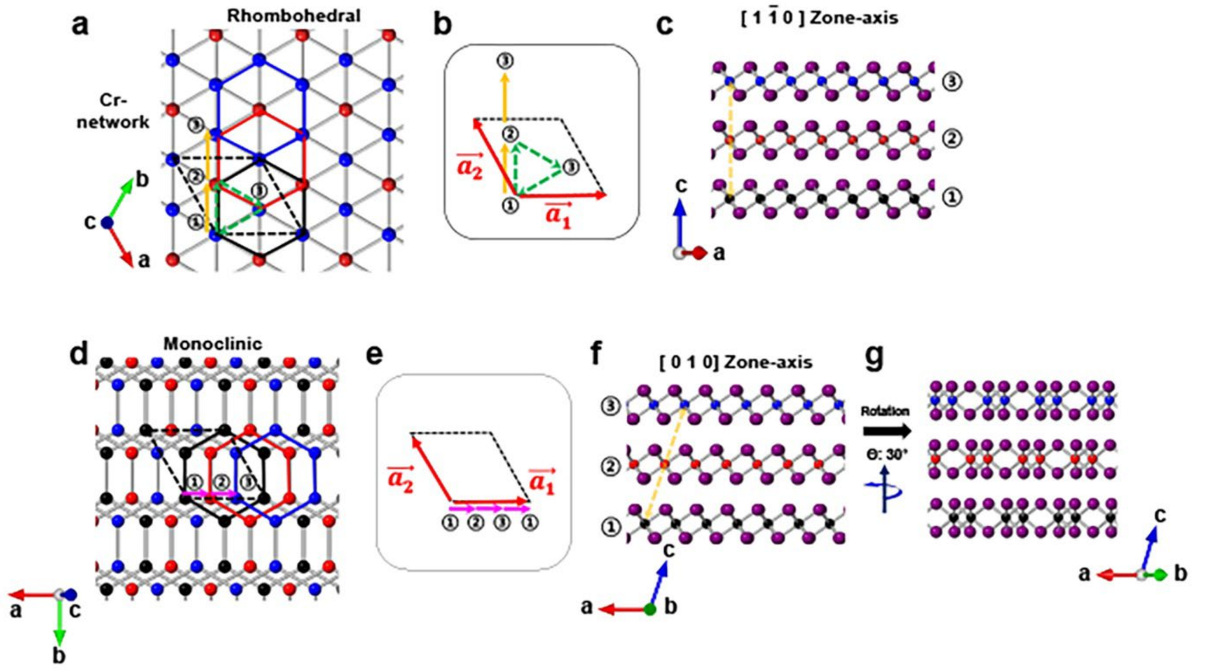


**Supplementary Figure 1. Stacking configurations in rhombohedral (low-temperature) vs. monoclinic (room-temperature) phases of $CrI_3$.** (a) Top-view schematics showing Cr positions in rhombohedral stacking. The arrows indicate the relative lateral shift between adjacent layers. (b) Relative lateral shift between adjacent layers in rhombohedral stacking configurations. The lateral shift of $(1/3)\vec{a}_1 + (2/3)\vec{a}_2$ (green dashed arrow or yellow arrow) is shown. (c) Rhombohedral structure viewed along y-axis, [010] zone axis. Cr positions in each layer show the same lateral position. (d) Top-view schematics showing Cr positions in monoclinic stacking. The arrows show the relative lateral shift between adjacent layers. (e) Relative lateral shift between adjacent layers in monoclinic stacking configurations. The lateral shift of $(1/3)\vec{a}_1$ is shown. (f) Monoclinic structure viewed along y-axis, [010] zone axis. Cr positions in each layer show the relative lateral shift. (g) Monoclinic structure view after 30-degree rotation with z-axis as a rotational axis.

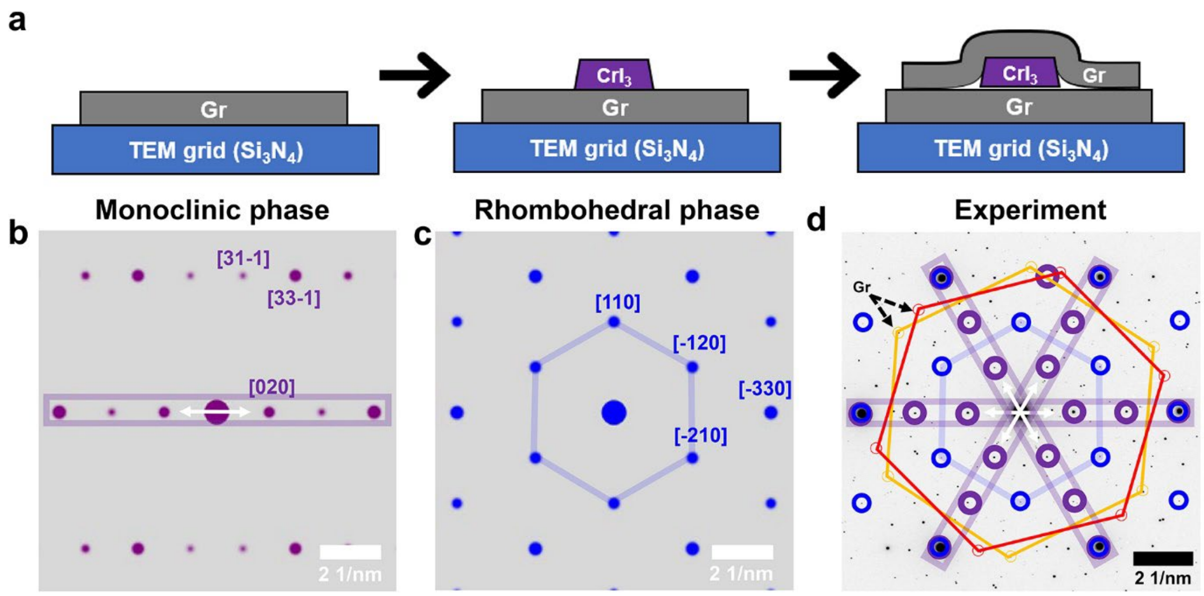


**Supplementary Figure 2. Plan-view analysis of $CrI_3$ TEM samples.** (a) Plan-view (top-view) TEM sample preparation process. Top and bottom thin graphite layers encapsulate exfoliated $CrI_3$ flakes. (b) Simulated SAED pattern of monoclinic $CrI_3$ (c) Simulated SAED pattern of rhombohedral stacking $CrI_3$. (d) Experimental SAED pattern of $CrI_3$. Diffraction signals from the top and bottom graphite flakes are marked with yellow and red hexagons. Extra peaks are also present due to double scattering between graphite (top or bottom) layers and $CrI_3$.

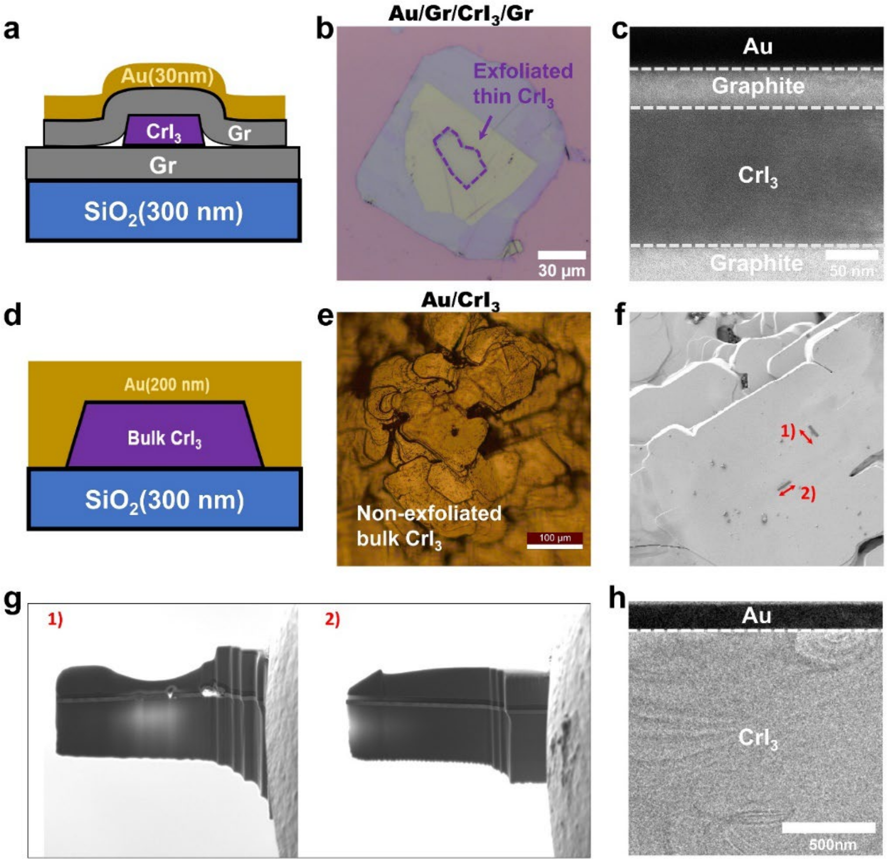


**Supplementary Figure 3. Cross-section TEM sample preparation.** (a) Schematic of cross-sectional $CrI_3$ TEM samples (exfoliated crystal). (b) Optical image of a graphite/$CrI_3$/graphite sample used for FIB processing (top view). (c) Low-magnification cross-sectional TEM image of a FIB-processed $CrI_3$ sample. (d) Schematic of cross-sectional $CrI_3$ TEM samples (bulk as-grown crystal). (e) Optical image of Au (200 nm) coated $CrI_3$ sample used for FIB processing (top view). (f) SEM image of FIB processing of $CrI_3$ crystal with two different directions (90-degree relative rotation). The marked lines indicate the direction of FIB sample preparation. (g) SEM images of cross- sectional sample after ion-milling. (h) Low-magnification cross-sectional TEM image of a FIB-processed as-grown $CrI_3$ sample.

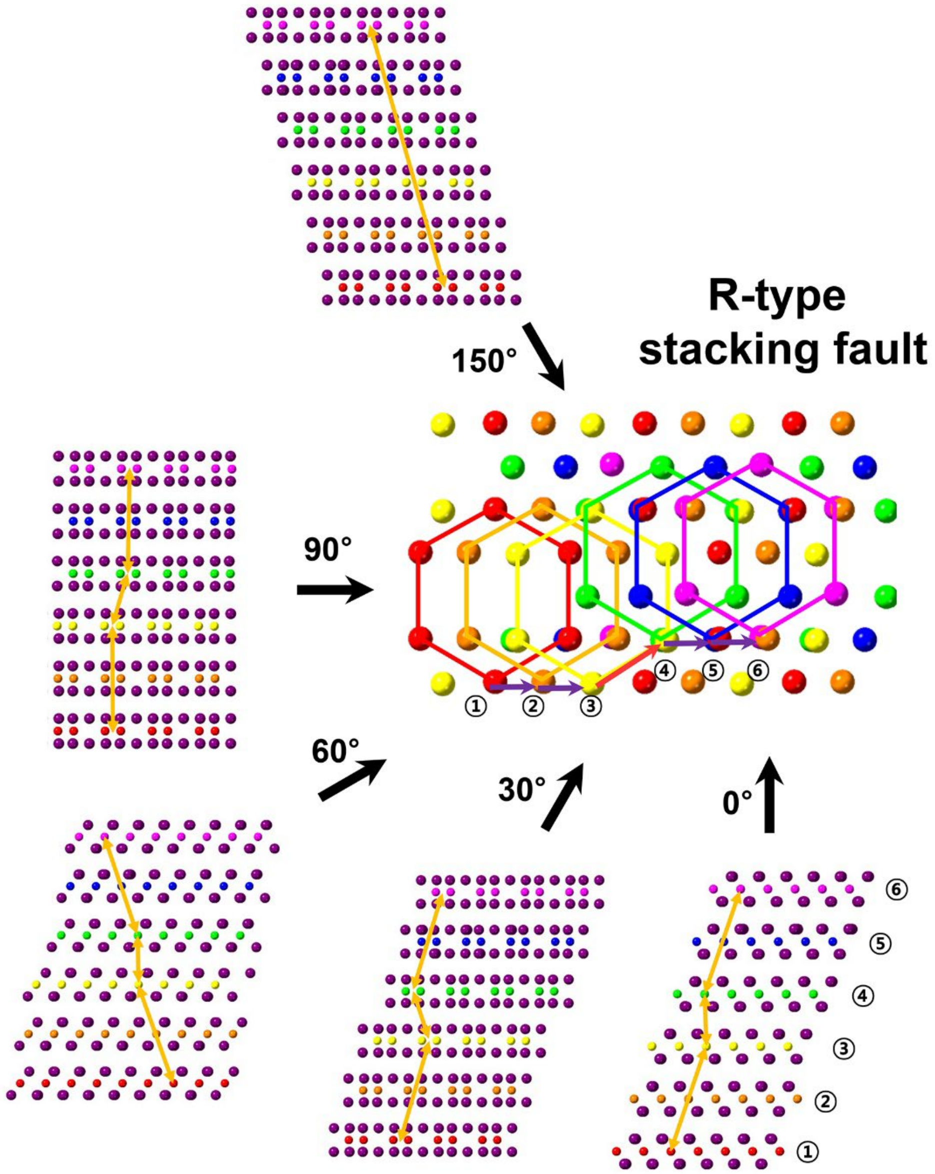


**Supplementary Figure 4. Rhombohedral-type (R-type) stacking fault in $CrI_3$.** Atomic structure models from different viewing directions are shown. There is an R-type stacking fault between the layer number 3 and 4.

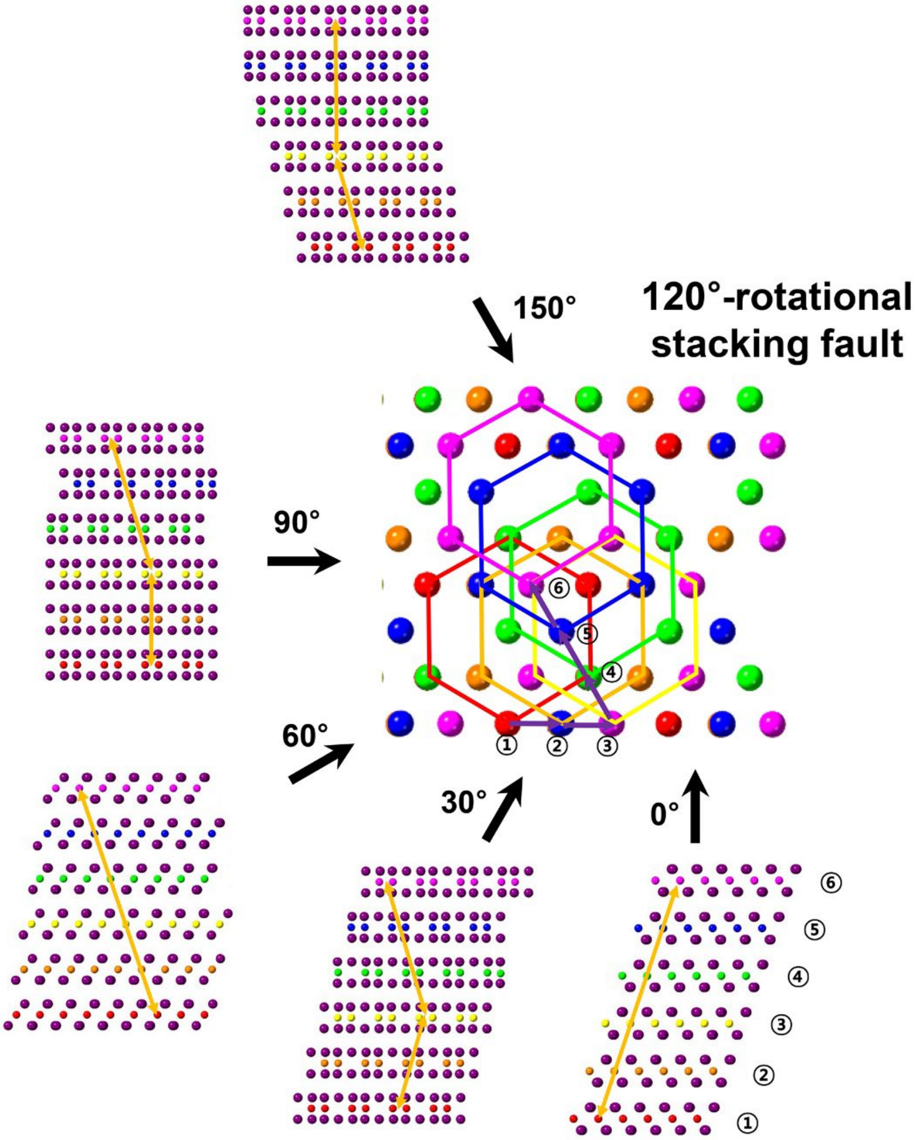


**Supplementary Figure 5. 120-degree twist stacking fault in $CrI_3$.** Atomic structure models from different viewing directions are shown. There is a 120-egree rotational stacking fault with the layer number 3.

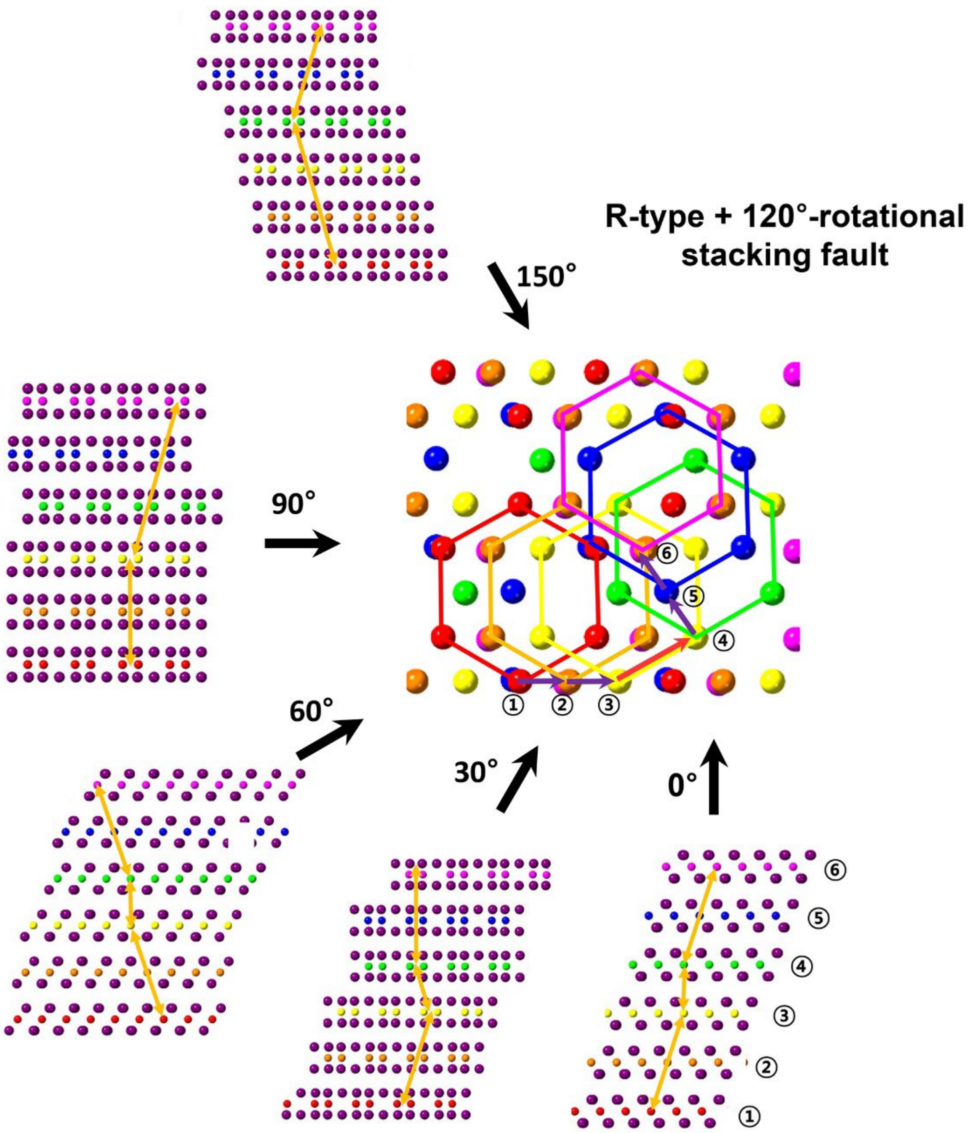


**Supplementary Figure 6. 120-degree twist⊕R-type fault in $CrI_3$.** Atomic structure models from different viewing directions are shown.

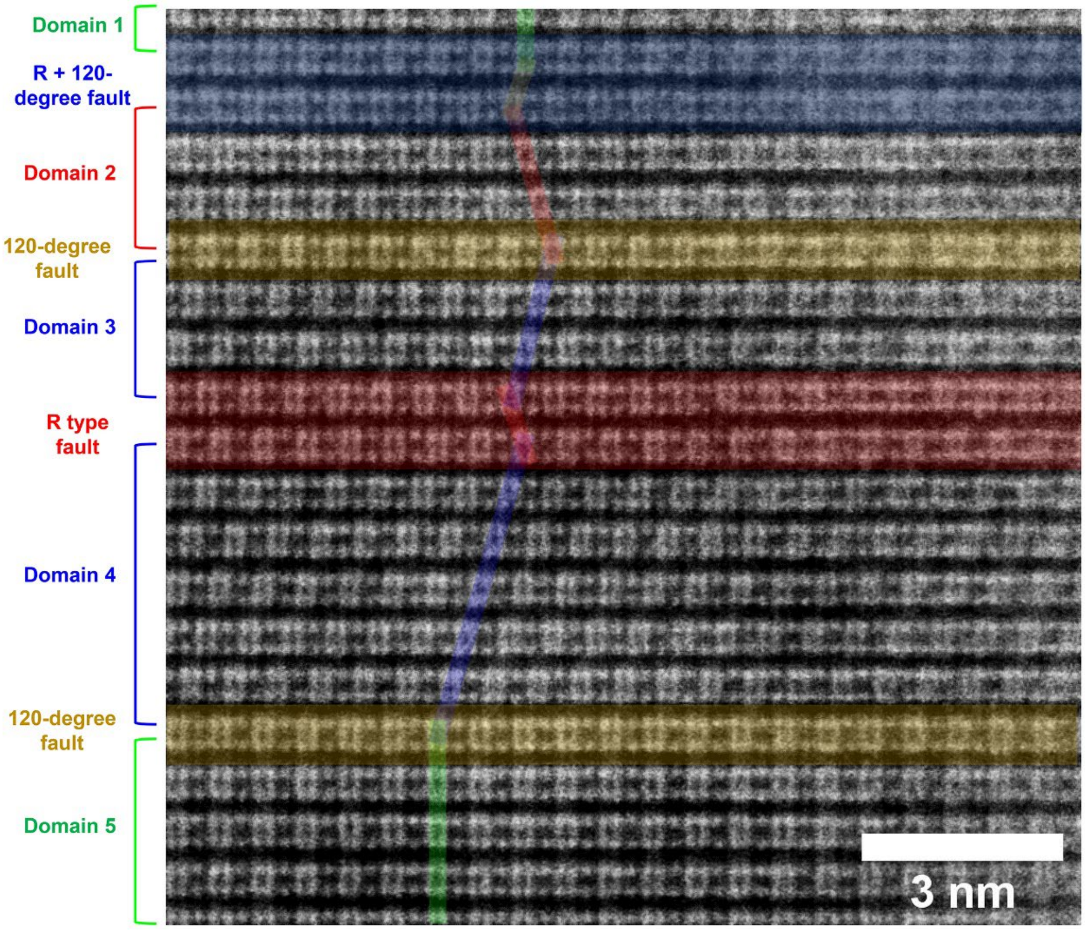


**Supplementary Figure 7. Extra HAADF-STEM image for analysis of stacking fault types and domain size distribution.** HAADF-STEM image with R-type fault, 120-degree twisted fault, and twisted⊕R stacking fault are shown in the image.

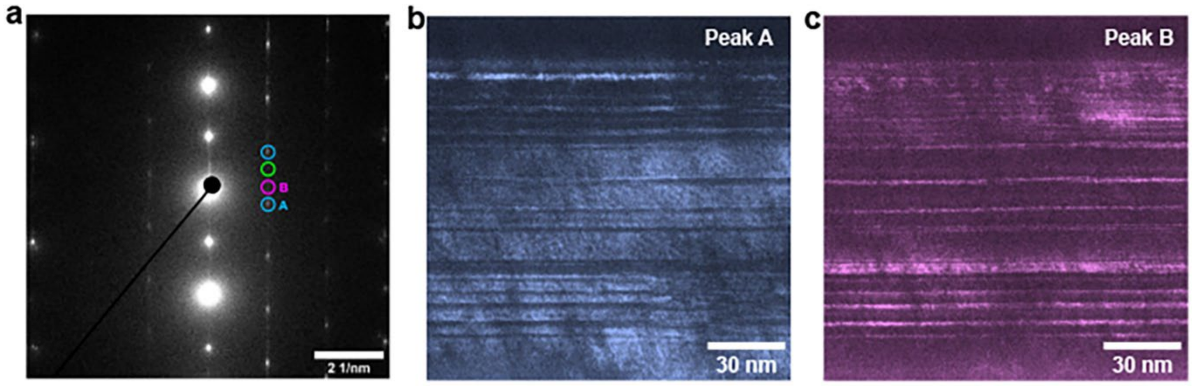


**Supplementary Figure 8. Extra DF imaging data from exfoliated samples.** (a) Experimental SAED. The marked diffraction peaks are used for DF imaging. (b) DF image of stacking domains with peak A labeled in panel a. (c) DF image of stacking domains with peak B labeled in panel a.

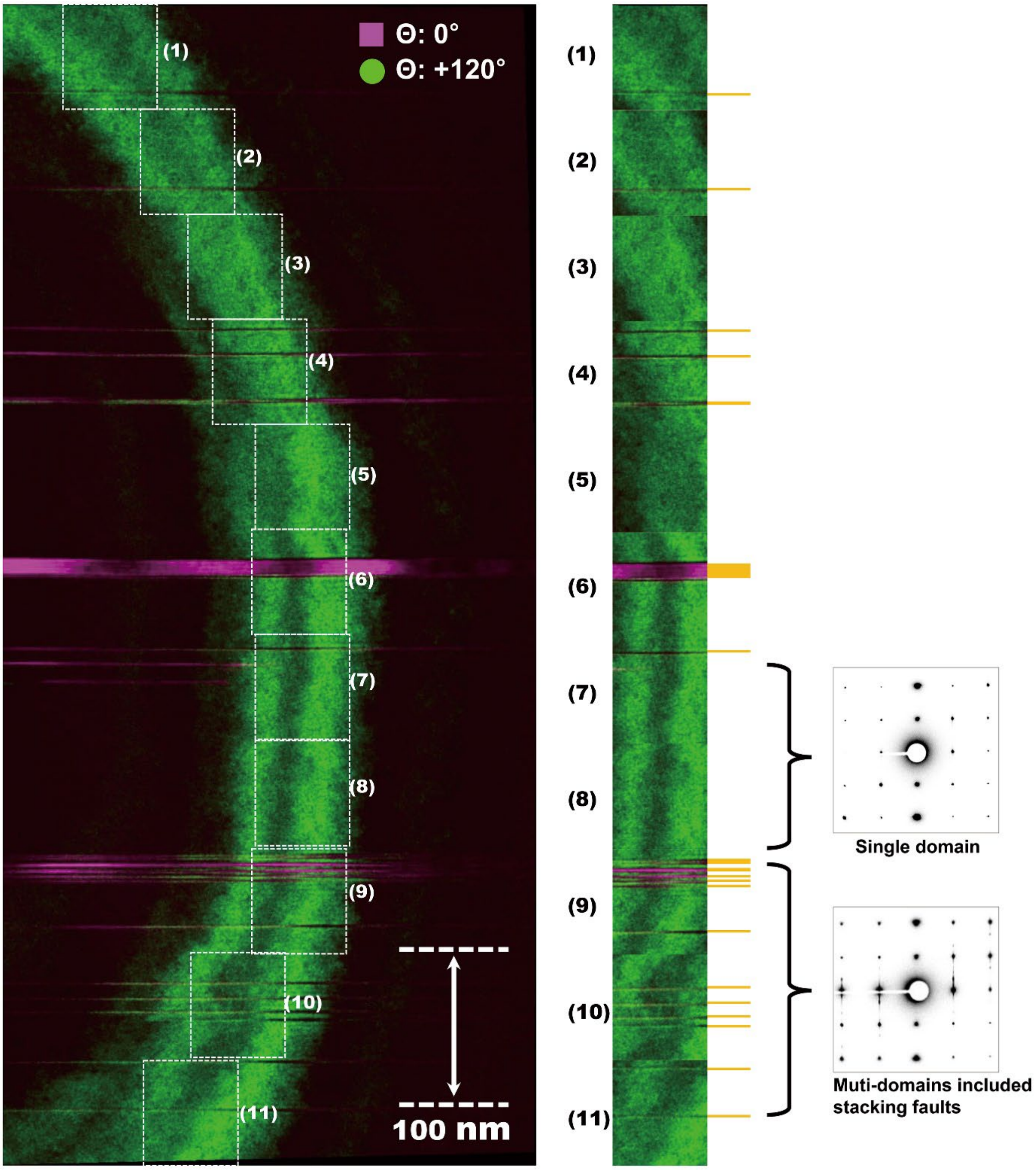


**Supplementary Figure 9. Dark field imaging of as-grown unexfoliated $CrI_3$**. The left image shows the color-overlaid DF image. The far right panels correspond to electron diffraction pattern from different regions of the sample.

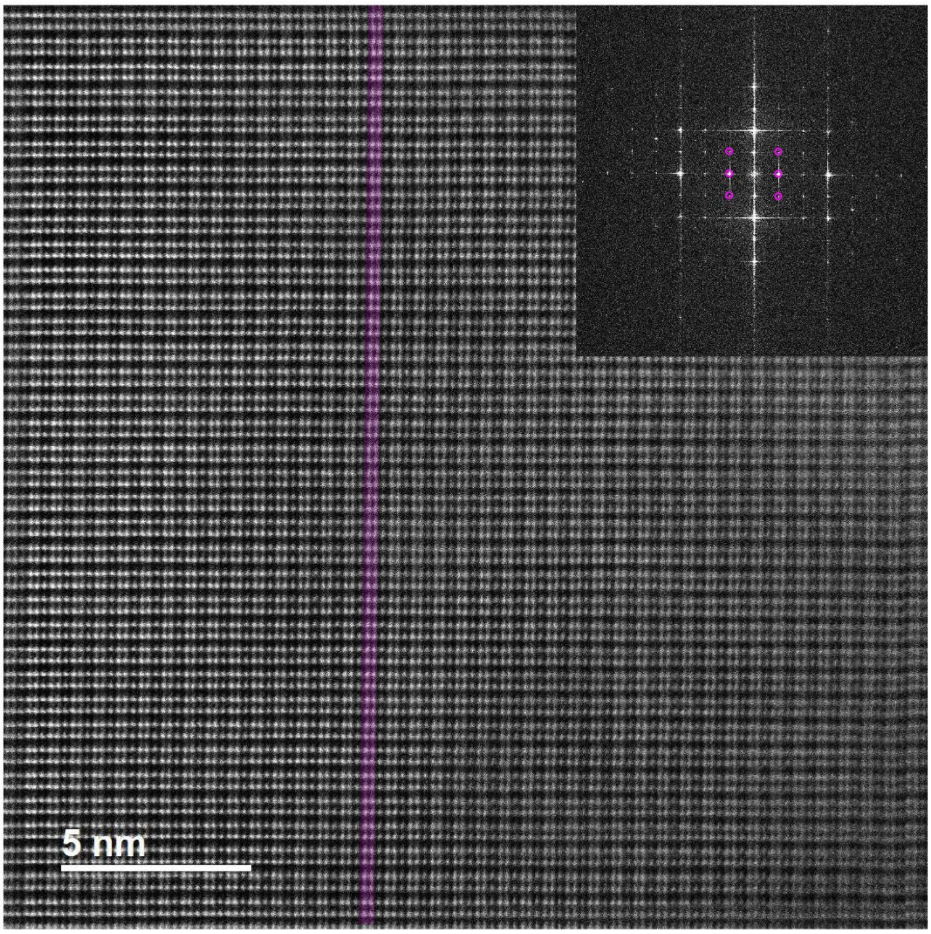


**Supplementary Figure 10. Extra cross-section HAADF-STEM image of as-grown unexfoliated $CrI_3$.** The field of view in the STEM image is of single-crystalline without a stacking fault. The vertical line indicates the Cr direction in the sample. The inset is the fast Fourier transform (FFT) of the image.

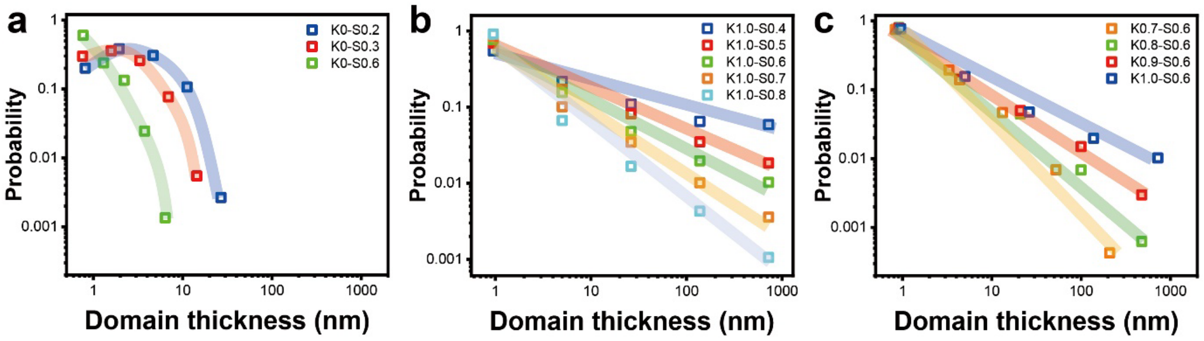


**Supplementary Figure 11. Simulation results for vertical domain size distribution.** We model the vertical layer-by-layer growth behavior where the stacking relation between $(n-1)^{\text{th}}$ and $n^{\text{th}}$ layers is either with stacking fault or without. The probability of a stacking fault occurrence for $n^{\text{th}}$ layer is given by $P(x) = S\,x^{-k}$, where $S$ and $k$ are constant values, and $x$ (integer) is the interlayer distance from a underneath nearest neighbor stacking fault. $k$ is the constant which determine the correlation regarding the location of stacking faults. (a) Simulated domain distribution results with $k = 0$ (random stacking faults). The domain size distribution falls exponentially and cannot explain the existence of big domains in experiments. (b) Simulated domain distribution results with $k = 1$ and various $S$ values. Higher $S$ values lead to an increased proportion of small domains and the change of distribution slope. (c) Simulated domain distribution results with $S = 0.6$ and various $k$ values. Higher values of $k$ results in the increased formation of bigger domains.

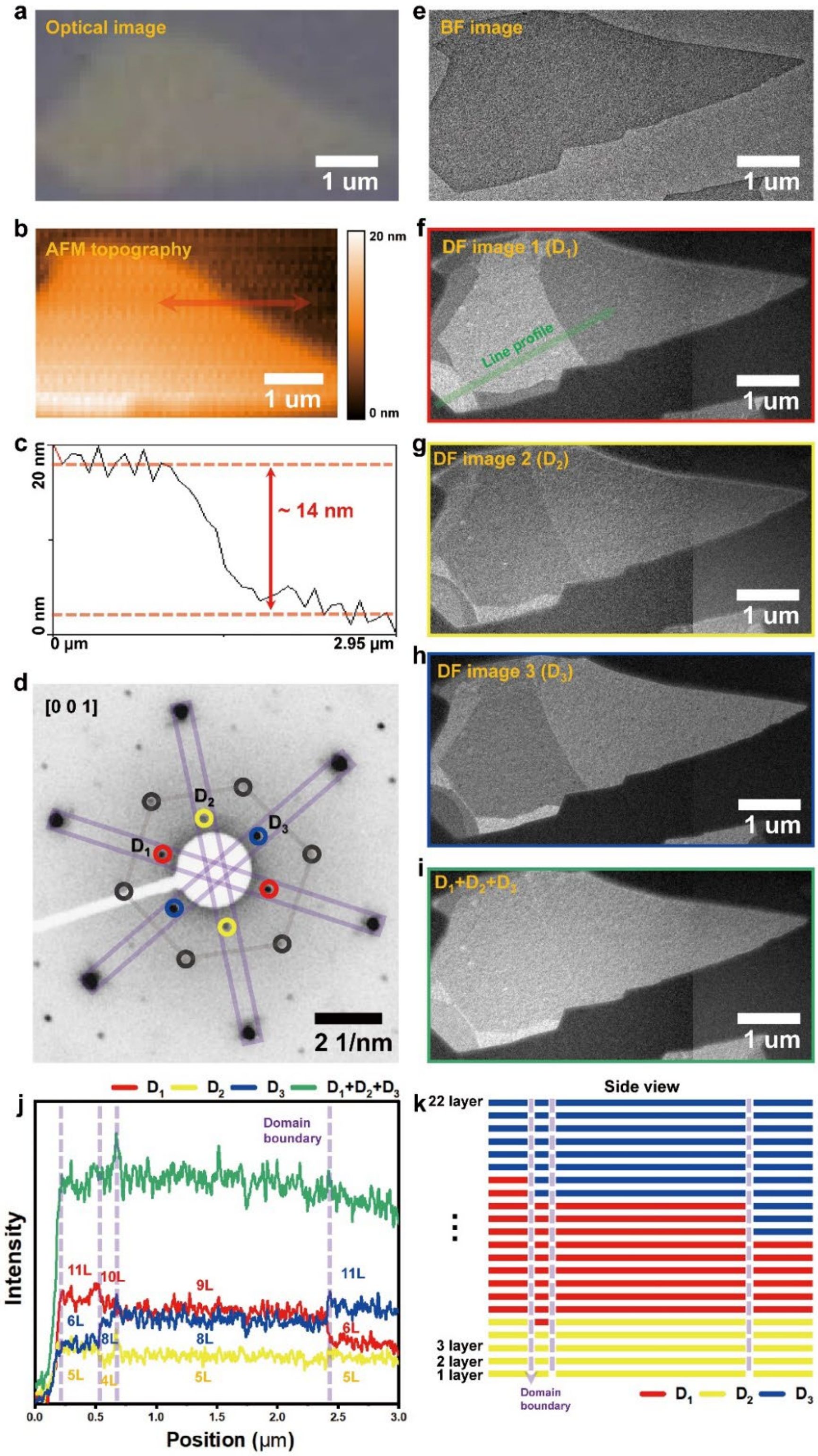


**Supplementary Figure 12. Visualization of stacking domains in plan-view $CrI_3$ samples of 14-nm thickness using dark-field TEM imaging.** (a) Optical image of a mechanically-exfoliated $CrI_3$ flake on $Si_3N_4$ membrane TEM grid (membrane thickness: 30 nm). (b) AFM topography image of the $CrI_3$ flake. The AFM measurement was performed inside a glovebox

filled with $N_2$. (c) The AFM line profile along the red line in panel b. The sample thickness is approximately 14 nm. (d) Electron diffraction pattern of the sample. The signals from three monoclinic variants with different sliding directions are marked with violet circles and labeled as $D_1$, $D_2$, and $D_3$. (e) Bright-field TEM image of the sample. (f – h) Dark-field TEM images obtained by selecting $D_1$, $D_2$, and $D_3$ peaks. (i) A total sum image combining panels f, g, and h. (j) Intensity profiles of the DF images and their sum across the green line in panel f. The local twisted domain thickness is estimated from the intensity in the DF images. The purple dashed lines indicate the location of domain boundaries. (k) A schematic representation of a proposed lateral twisted domain structure derived from the line intensity profiles of DF images.

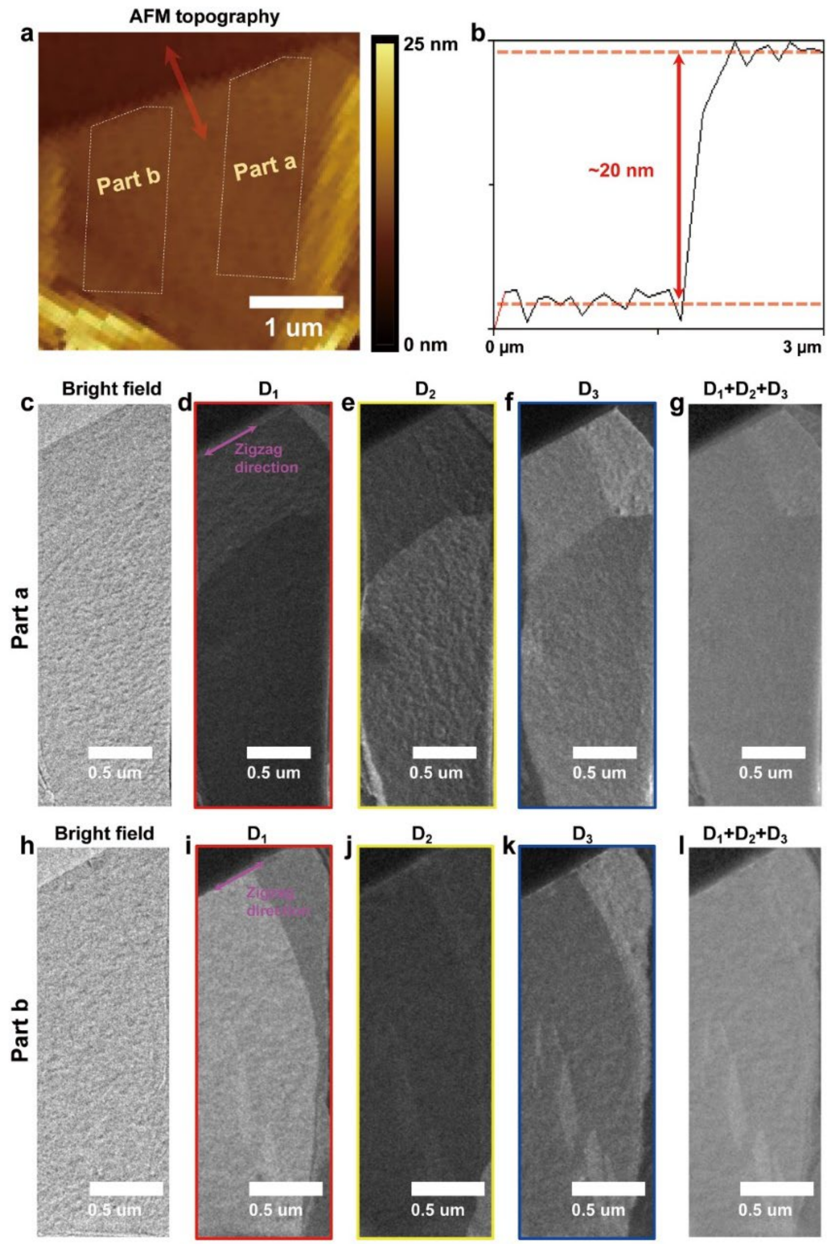


**Supplementary Figure 13. Visualization of stacking domains in plan-view $CrI_3$ samples of 20-nm thickness using dark-field TEM imaging.** (a) AFM topography image of the $CrI_3$ flake. (b) The AFM line profile along the red line in panel a. The sample thickness is approximately 20 nm. (c) Bright-field TEM image of the sample in region a. (d– f) Dark-field TEM images obtained by selecting $D_1$, $D_2$, and $D_3$ peaks. The sample edge is aligned with the zigzag lattice direction as shown in panel d. (g) A total sum image combining panels d, e, and f. (h) Bright-field TEM image of the sample in region b. (i– k) Dark-field TEM image obtained by selecting $D_1$, $D_2$, and $D_3$. (l) A total sum image combining panels i, j, and k.

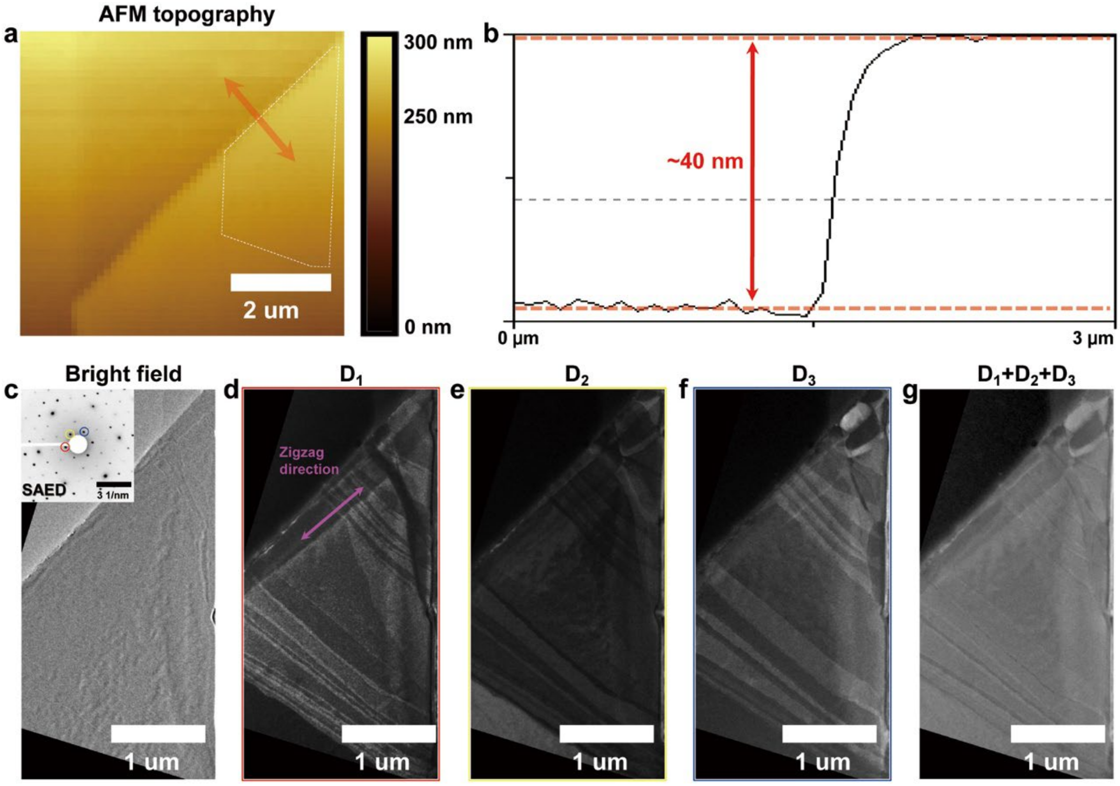


**Supplementary Figure 14. Visualization of stacking domains in plan-view $CrI_3$ samples of 40-nm thickness using dark-field TEM imaging.** (a) AFM topography image of the $CrI_3$ flake. (b) The AFM line profile along the red line in panel a. The sample thickness is approximately 40 nm. (c) Bright-field TEM image of the sample. The inset is electron diffraction from the sample. (d– f) Dark-field TEM images obtained by selecting $D_1$, $D_2$, and $D_3$ peaks. The sample edge is aligned with the zigzag lattice direction as shown in panel d. (g) A total sum image combining panels d, e, and f.

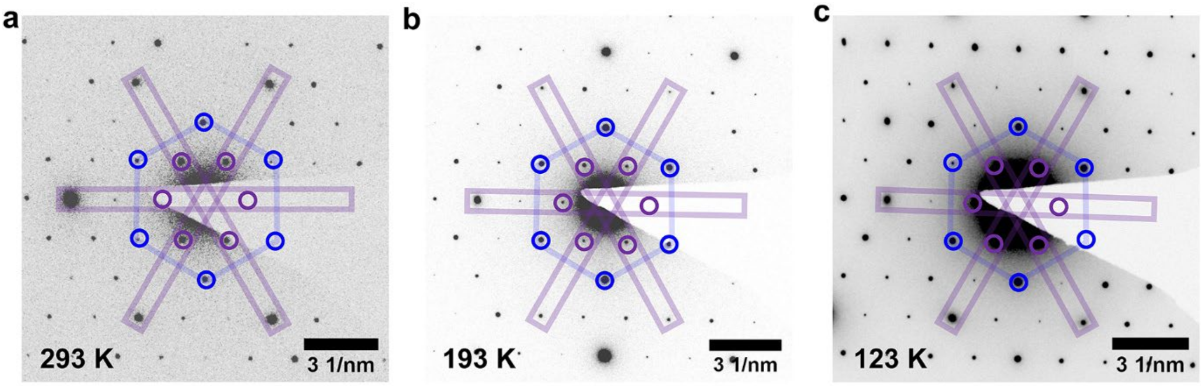


**Supplementary Figure 15. Representative SAED of monoclinic $CrI_3$ from out-of-plane direction at different temperatures.** (a), (b) and (c) SAED obtained at room temperature, 193 K and 123 K, respectively.

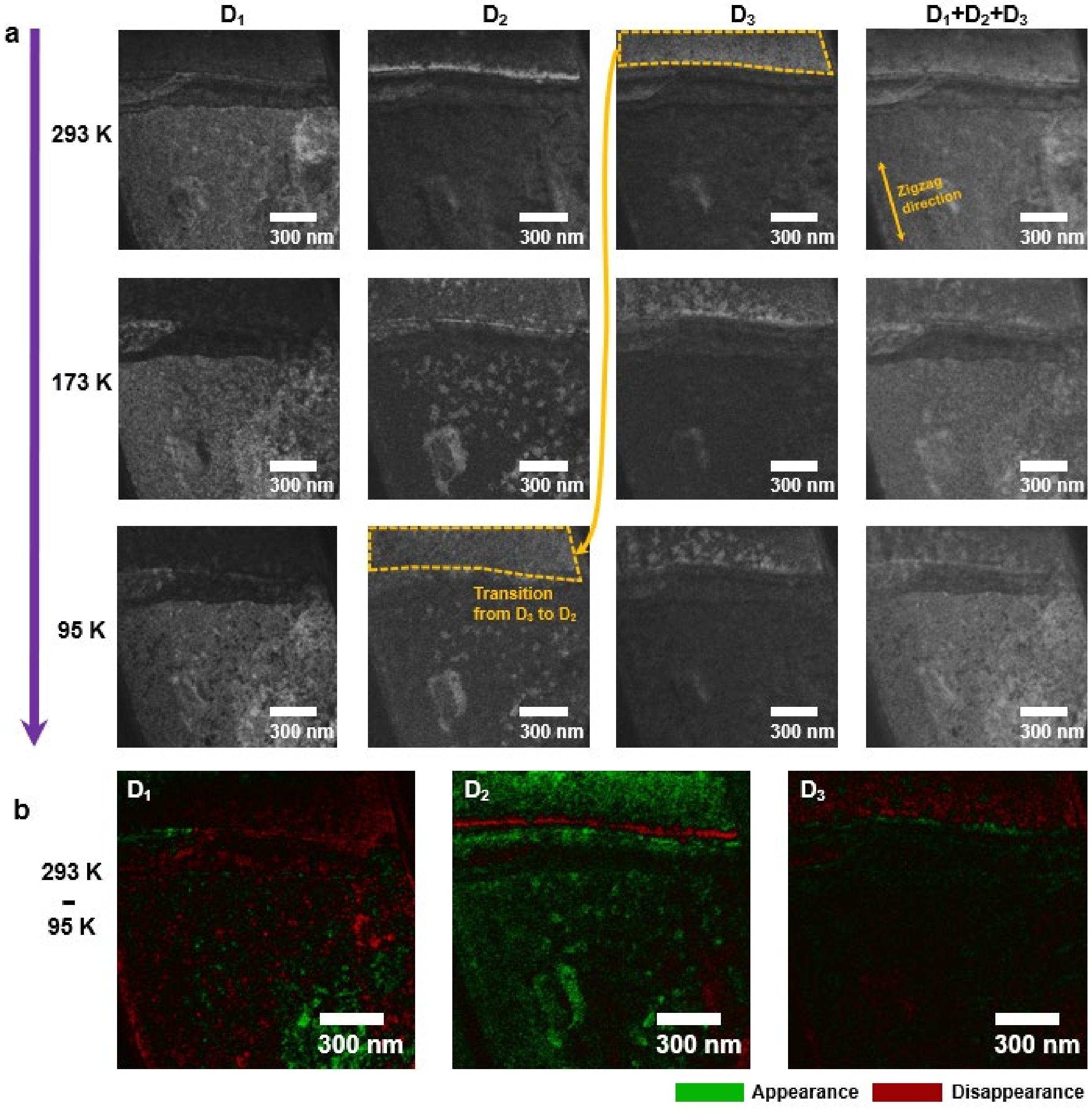


**Supplementary Figure 16. Observation of lateral domain changes in $CrI_3$ at low temperatures using DF imaging.** (a) Observation of lateral domain changes with temperature variation. While the $D_1$ region remains largely unchanged with temperature, a transition from the orange dashed region of $D_3$ to the $D_2$ region is observed as the temperature decreases. (b) An image illustrating disappeared and emerged domains using color indicators. Red areas indicate domains that vanished as the temperature dropped from room temperature to a lower temperature, while green areas indicate newly formed domains.

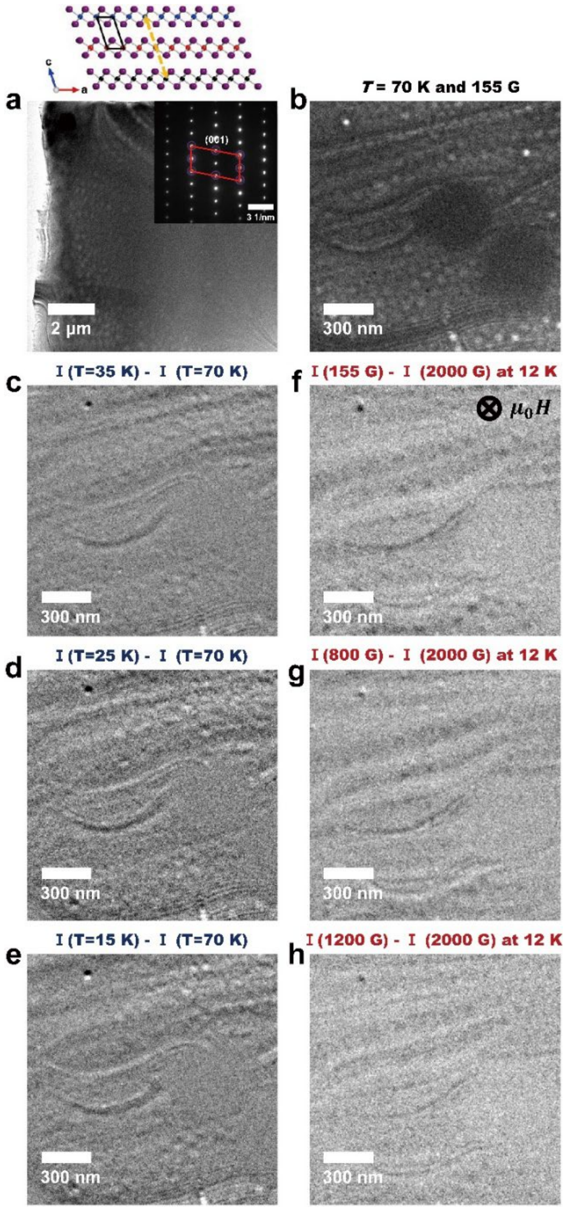


**Supplementary Figure 17. Lorentz TEM imaging of $CrI_3$ sample at low temperatures.** (a) TEM image of a FIB-processed $CrI_3$ sample along the [010] zone axis. The inset shows the electron diffraction signal showing the monoclinic phase of $CrI_3$. (b) Lorentz TEM image at 70 K under the residual magnetic field of 155 G. Under out-of-focus imaging required for Lorentz TEM, the contrast features such as small particles possibly due to surface contamination or surface defects were observed. To isolate the magnetic contrast and reduce the effect of background, the image contrast differences compared to that measured at high temperatures or at high applied field of 2000 G were monitored. (c-e) LTEM images during the cool-down from 70 K to 15 K at 155 G. (c) 35 K, (d) 25 K and (e) 15 K. (f-h) Lorentz TEM images during in-situ applied field of (b) 155 G, (c) 800 G and (d) 1200 G at 12 K.

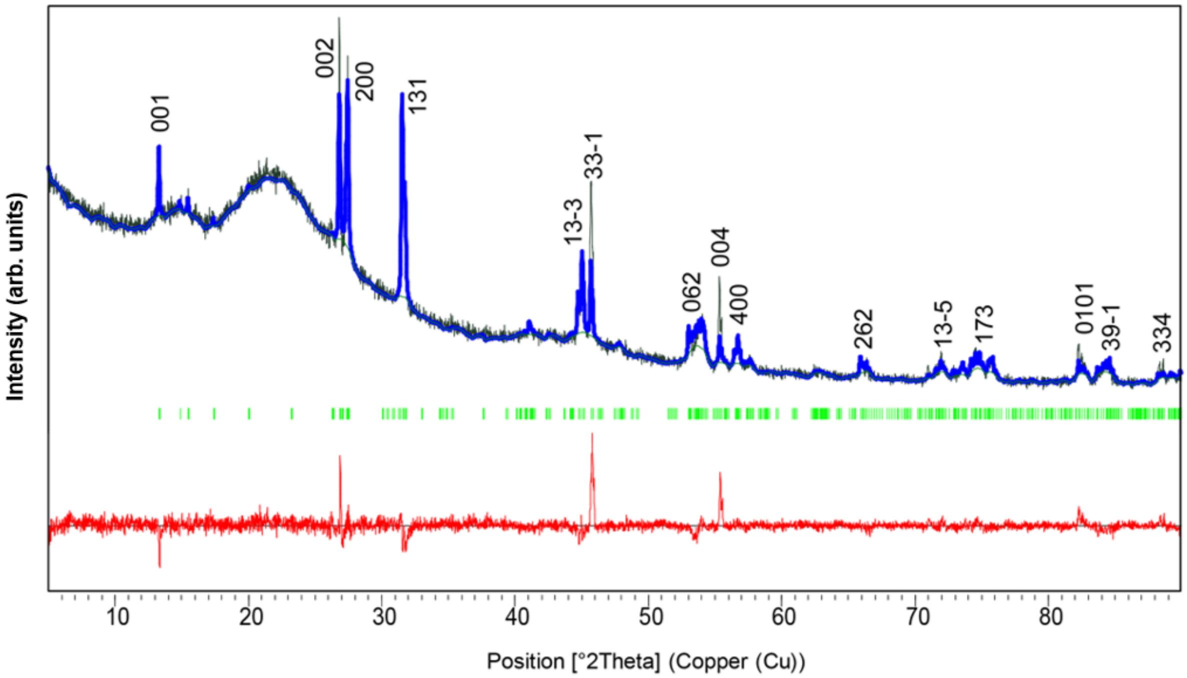


**Supplementary Figure 18. The XRD pattern and unit cell refinement of $CrI_3$ single crystals.** $CrI_3$ crystal structure was characterized by X-ray diffraction (XRD). Supplementary Figure 10 shows the XRD of the $CrI_3$ single crystals obtained employing PANalytical Empyrean X-ray diffractometer. X-Rays diffraction analysis was performed on a sample of single-crystalline $CrI_3$ by loading the material into a capillary and sealing it inside the glove box. The powder pattern of the sample is consistent with the monoclinic $AlCl_3$-type structure (C2/m) reported for $CrI_3$. In the XRD spectrum, no evidence of any other phases was detected indicating that the product is of high purity.

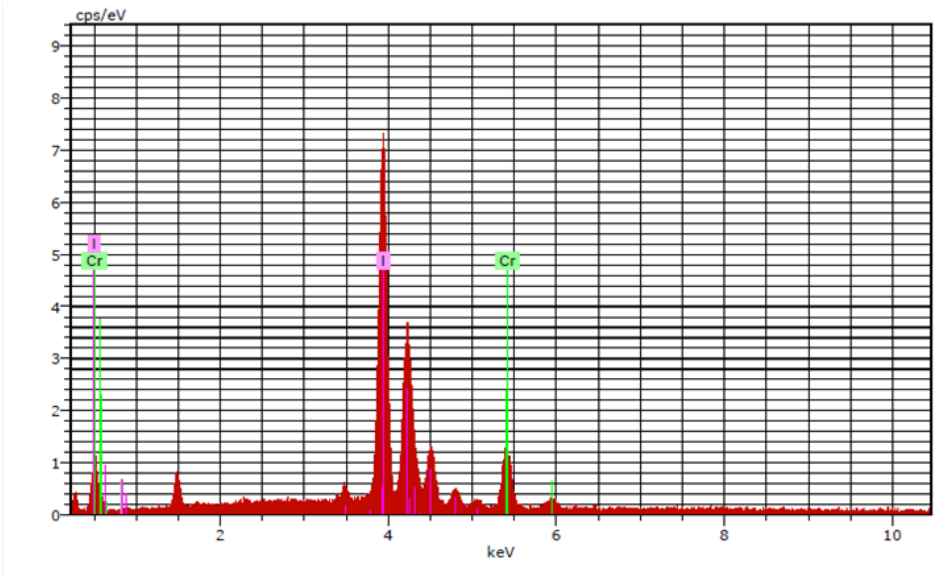


**Supplementary Figure 19. The EDAX spectrum of the as-grown $CrI_3$ single crystals.**

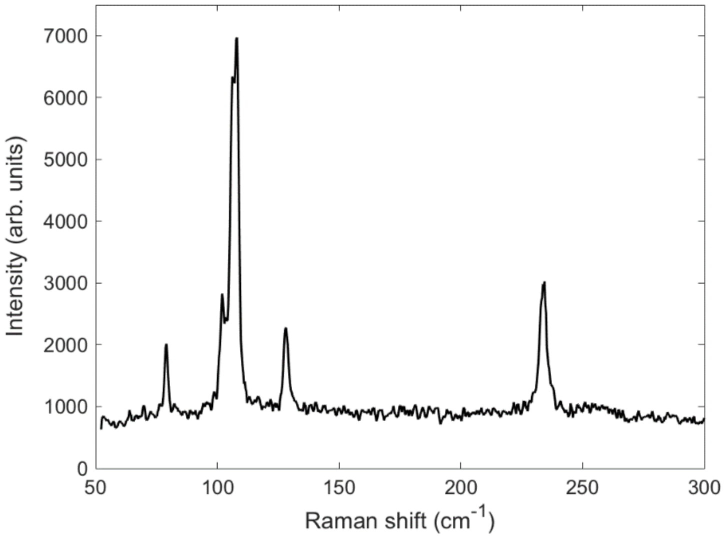


**Supplementary Figure 20. Raman spectrum of bulk $CrI_3$ flake.** $CrI_3$ bulk layers have been characterized by Raman spectroscopy after their mechanical exfoliation to verify their pristine quality. Raman characterization has been performed using HORIBA (LabRAM HR Evolution) Raman spectrometer under a 532 nm excitation laser. $CrI_3$ crystals display a series of peaks at frequencies of 78, 100−110, 128, and 234 $cm^{-1}$ similar to results reported in the literature confirming the quality of $CrI_3$ layers.

**Supplementary Table 1. $CrI_3$ samples investigated by cross-sectional TEM.**

| Sample preparation | Sample number | Thickness |
|---|---|---|
| Exfoliated samples | Sample 1 | ~ 100 nm |
| | Sample 2 | ~ 130 nm |
| | Sample 3 | ~ 180 nm |
| | Sample 4 | ~ 430 nm |
| As-grown unexfoliated samples | Sample 5 | ~ 2 um |
| | Sample 6 | ~ 4.4 um |
| | Sample 7 | ~ 5 um |

**Supplementary Table 2. Elemental analysis of $CrI_3$ by EDX**

| Elements | Weight % | Atomic % |
|---|---|---|
| **Cr** | 11.73 | 24.49 |
| **I** | 88.27 | 75.51 |

The chemical composition of as-grown $CrI_3$ single crystals was determined by energy dispersive analysis of X-ray (EDX) technique using Hitachi S4800 for confirming stoichiometry. The obtained spectrum is shown in Supplementary Figure 11. The atomic and weight % obtained using EDX is tabulated in Supplementary Table 2. The data clearly states that the as-grown CVT single crystals have a chemical composition of $CrI_3$ and they are free from any impurity.
